# Implication of REDD1 in the activation of inflammatory pathways

**DOI:** 10.1038/s41598-017-07182-z

**Published:** 2017-08-01

**Authors:** Faustine Pastor, Karine Dumas, Marie-Astrid Barthélémy, Claire Regazzetti, Noémie Druelle, Pascal Peraldi, Mireille Cormont, Jean-François Tanti, Sophie Giorgetti-Peraldi

**Affiliations:** 1 Université Nice Côte d’Azur, Inserm U1065, C3M, Team Cellular and Molecular Physiopathology of Obesity, Nice, France; 2 Université Nice Côte d’Azur, Inserm U1091, CNRS U7277, iBV, Team “Stem cells and differentiation”, Nice, France; 3 Present Address: Université Nice Côte d’Azur, Inserm U1065, C3M, Team “ Study of the melanocytic differentiation applied to vitiligo and melanoma: from the patient to the molecular mechanisms”, Nice, France; 4 Present Address: Université Nice Côte d’Azur, Inserm U1091, CNRS U7277, iBV, Team Diabetes genetic team, Nice, France

## Abstract

In response to endotoxemia, the organism triggers an inflammatory response, and the visceral adipose tissue represents a major source of proinflammatory cytokines. The regulation of inflammation response in the adipose tissue is thus of crucial importance. We demonstrated that Regulated in development and DNA damage response-1 (REDD1) is involved in inflammation. REDD1 expression was increased in response to lipopolysaccharide (LPS) in bone marrow derived macrophages (BMDM) and in epidydimal adipose tissue. Loss of REDD1 protected the development of inflammation, since the expression of proinflammatory cytokines (TNFα, IL-6, IL-1β) was decreased in adipose tissue of REDD1^−/−^ mice injected with LPS compared to wild-type mice. This decrease was associated with an inhibition of the activation of p38MAPK, JNK, NF-κB and NLRP3 inflammasome leading to a reduction of IL-1β secretion in response to LPS and ATP in REDD1^−/−^ BMDM. Although REDD1 is an inhibitor of mTORC1, loss of REDD1 decreased inflammation independently of mTORC1 activation but more likely through oxidative stress regulation. Absence of REDD1 decreases ROS associated with a dysregulation of Nox-1 and GPx3 expression. Absence of REDD1 in macrophages decreases the development of insulin resistance in adipocyte-macrophage coculture. Altogether, REDD1 appears to be a key player in the control of inflammation.

## Introduction

Inflammation is the response of the innate immune system to pathogens or injury and is due to secretion of proinflammatory cytokines. Inflammation can be induced by the activation of toll-like receptors (TLR) by pathogen-associated molecular patterns (PAMPs), such as lipopolysaccharide (LPS). Increased LPS-induced endotoxemia activates TLR4 and downstream pathways such as MAP kinases and NF-κB signaling pathways to promote proinflammatory cytokine secretion, such as IL-1β. IL-1β is produced in response to infection through the activation of the multiprotein platform NLRP3 inflammasome and caspase-1^1, 2^.

The visceral adipose tissue is a major source of proinflammatory cytokines production during acute systemic inflammation. Indeed, LPS injection in mice leads to an increase expression and secretion of proinflammatory cytokines including TNFα, IL-1β and IL-6. These proinflammatory cytokines are produced by cells from the stromal vascular fraction such as macrophages, rather than adipocytes^3, 4^.

Recently, it has been demonstrated that REDD1 (Regulated in development and DNA damage response 1) could be involved in LPS-induced inflammation. Indeed, intratracheal administration of LPS stimulates pulmonary inflammation with an upregulation of REDD1 protein expression, and loss of REDD1 protects lung inflammation induced by LPS^5^. REDD1 is a stress-induced protein, whose expression is finely regulated by the activation of specific transcription factors, including HIF-1, p53 or ATF4-C/EBPβ^6–9^. REDD1 acts as an inhibitor of mTORC1 by regulating the activity of TSC2 through two distinct mechanisms: the sequestration of 14-3-3 proteins away from TSC2^10^ and/or the PP2A-dependent dephosphorylation of PKB, both mechanisms leading to an increase of TSC2 activity and the subsequent inhibition of mTORC1^11^.

We have previously demonstrated that REDD1 is implicated in insulin signaling pathway. Indeed, insulin stimulates REDD1 expression by inhibiting its degradation through a MEK-dependent pathway^12^ and by activating its transcriptional regulation by HIF-1 transcription factor^13^. By controlling mTORC1 activity, REDD1 regulates insulin signaling pathway. Downregulation of REDD1 expression decreases insulin signaling pathway^12, 14, 15^, which is restored after the inhibition of mTORC1 with rapamycin^12, 14^.

REDD1 is also involved in neurodegenerative diseases such as Alzheimer and Parkinson disease where its expression is increased and correlates with mTORC1 inhibition and neuronal cell death^16–18^. In contrast, hyperactivation of mTORC1 is often found in cancer and has been associated with decreased REDD1 expression^7, 9, 10, 19^. Finally, REDD1 participates to glucocorticoid signaling and loss of REDD1 protects mice from muscle atrophy induced by dexamethasone^20^.

In addition of these cellular effects, REDD1 seems to be involved in inflammation because of its implication in LPS-induced lung inflammation^5^. Moreover, overexpression of REDD1 increases lung inflammation of mice exposed to tobacco smoke through the activation of NFκB activity, whereas loss of REDD1 protects the mice from pulmonary injury^21^.

The aim of the present study is to decipher the implication of REDD1 in the activation of inflammation. We demonstrate that loss of REDD1 inhibited the activation of p38 MAPK, JNK and NF-κB in response to LPS stimulation, as well as the activation of NLRP3 inflammasome and IL1β production in bone marrow derived macrophages or in adipose tissue explants from REDD1^−/−^ mice. These defects are associated with a reduced oxidative stress. Our results suggest that REDD1 is an important regulator of inflammation.

## Results

### Absence of REDD1 decreased inflammation induced by LPS injection in mice

Endotoxemia induced by LPS injection is known to stimulate the production of pro-inflammatory cytokines in adipose tissue^4^. Since REDD1 has been implicated in the activation of proinflammatory pathways in epithelial cells, we evaluated the effect of knockout of REDD1 on LPS-induced cytokines expression in adipose tissue.

**Figure 1:**
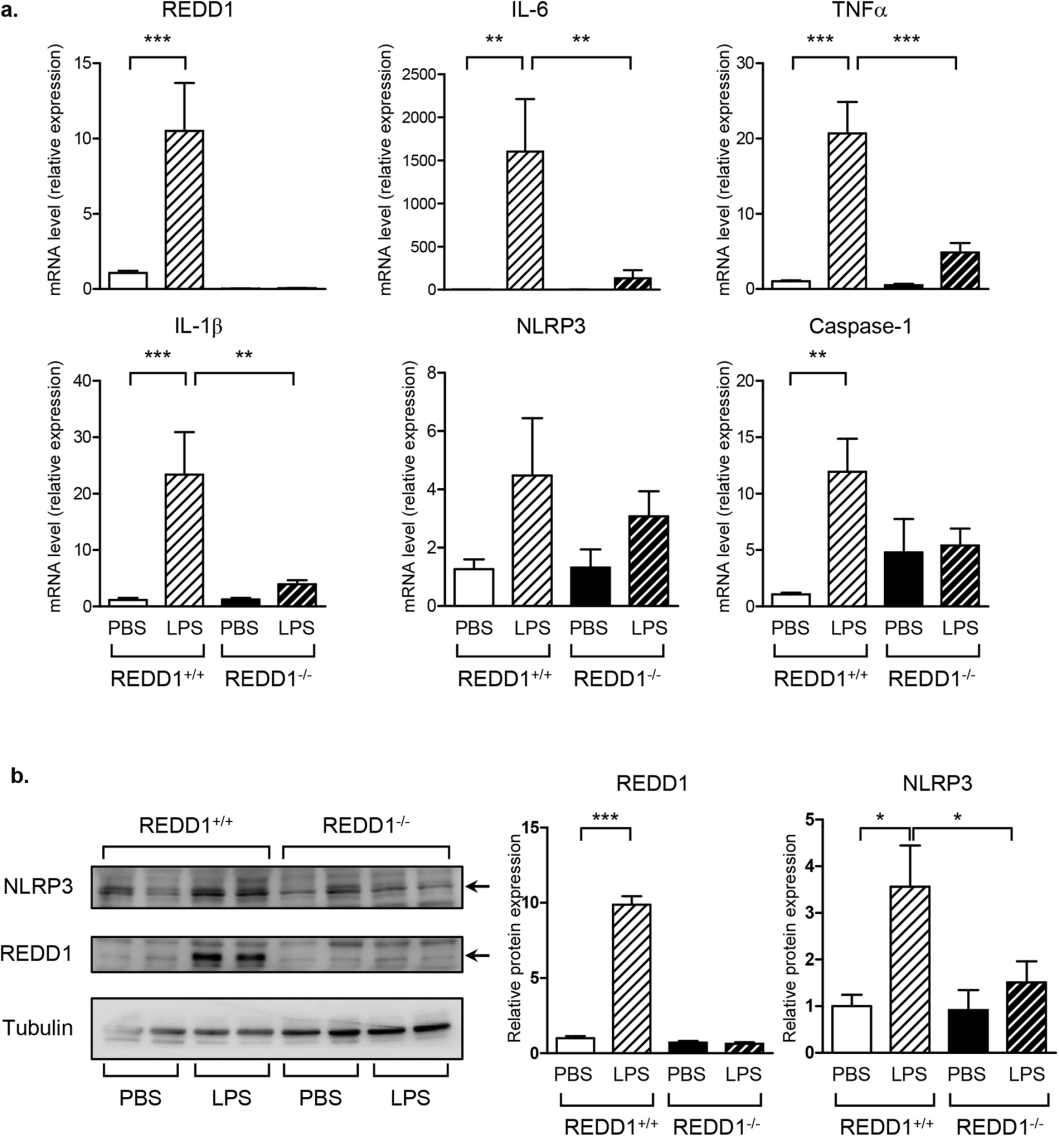
Inflammation was decreased in adipose tissue of REDD1^−/−^ mice injected with LPS. REDD1^+/+^ and REDD1^−/−^ mice were injected intraperitoneally with LPS (2 µg/g of body weight). After 5 hours, epididymal adipose tissue were recovered and (**a**) mRNA expression was determined by quantitative RT-PCR (n = 3 independent experiments with a total of 13 mice/group) and (**b**) protein expression was determined by immunoblots. Quantification of relative expression of NLRP3 and REDD1 is shown. (n = 4 mice/group) *p < 0.05; **p < 0.01, ***p < 0.0001.

REDD1^+/+^ and REDD1^−/−^ mice were subjected to intraperitoneal injection of LPS and epidydimal adipose tissue were collected and analyzed. LPS induced REDD1 mRNA and protein expression in wild-type mice (Fig. 1a and b). Moreover, LPS significantly stimulated the mRNA expression of proinflammatory factors such as IL-6, TNFα and IL-1β in REDD1^+/+^ mice. Absence of REDD1 impaired the expression of these cytokines in response to LPS (Fig. 1a). Since IL-1β expression is regulated by the activation of NLRP3 inflammasome, we studied the regulation of NLRP3 and caspase-1 expression in response to LPS injection. LPS stimulated the expression of caspase-1 mRNA, and NLRP3 mRNA and protein in adipose tissue in wild-type mice (Fig. 1a and b). In contrast, in REDD1^−/−^ mice, LPS failed to stimulate NLRP3 and caspase-1 expression in adipose tissue.

### REDD1 regulates the activation of NLRP3 inflammasome

**Figure 2:**
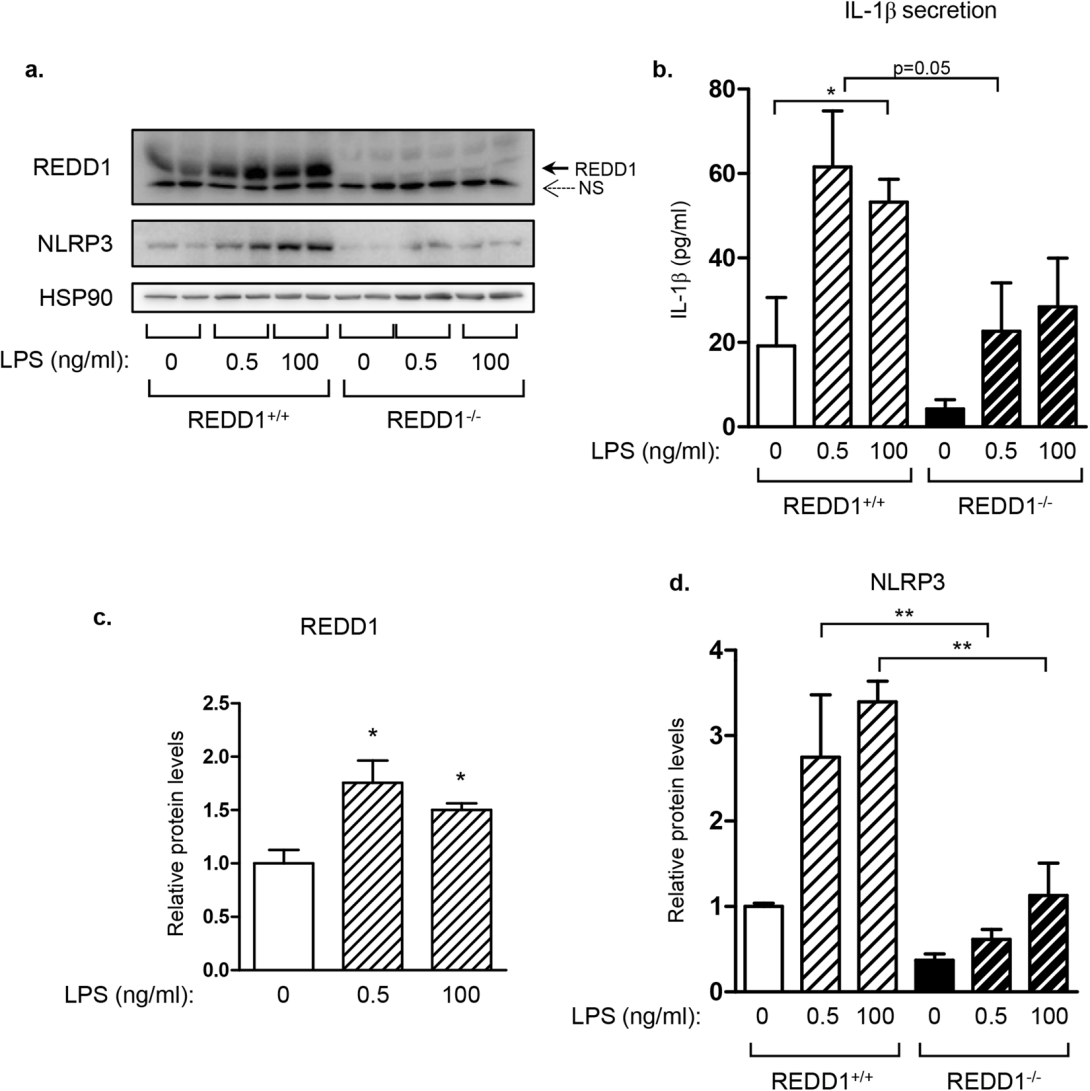
Induction of NLRP3 expression and secretion of IL-1β were inhibited in explants of adipose tissue. Adipose tissue explants isolated from REDD1^+/+^ and REDD1^−/−^ mice were stimulated for 5 hours with LPS (0.5 or 100 ng/ml) followed by a treatment with ATP (5 mM) for 45 minutes. (**a**) Lysates were analyzed by immunoblots with indicated antibodies. (**b**) IL-1β concentration was determined by elisa test in the culture supernatant (n = 3 independent experiments in duplicate). (**c** and **d**) Quantification of relative expression of REDD-1 and NLRP3 is shown (n = 3 independent experiments in duplicate). *p < 0.05; **p < 0.01.

Next, we investigated whether inflammasome activation in adipose tissue is regulated by REDD1. Assembly of NLRP3 inflammasome complex results in the pro-caspase-1 activation and pro-IL1β maturation. Activation of NLRP3 inflammasome required two signals: the priming which correspond to NF-κB-induced NLRP3 expression and the assembly of the complex triggered by ATP, bacterials toxins or lipid particles^1^. Epidydimal adipose tissue explants were prepared from REDD1^+/+^ and REDD1^−/−^ mice and treated with LPS for 5 hours followed by a treatment with ATP for 45 minutes to fully activate NLRP3 inflammasome (Fig. 2). In wild-type explants, LPS stimulated REDD1 and NLRP3 expression in a dose-dependent manner which was significantly reduced in REDD1^−/−^ explants (Fig. 2a,c and d). NLRP3 activation leads to the maturation and secretion of IL-1β. Indeed, LPS/ATP stimulation induced IL-1β secretion in REDD1^+/+^ adipose tissue explants, and this production of IL-1β was decreased in REDD1^−/−^ adipose tissue explants (Fig. 2b).

**Figure 3:**
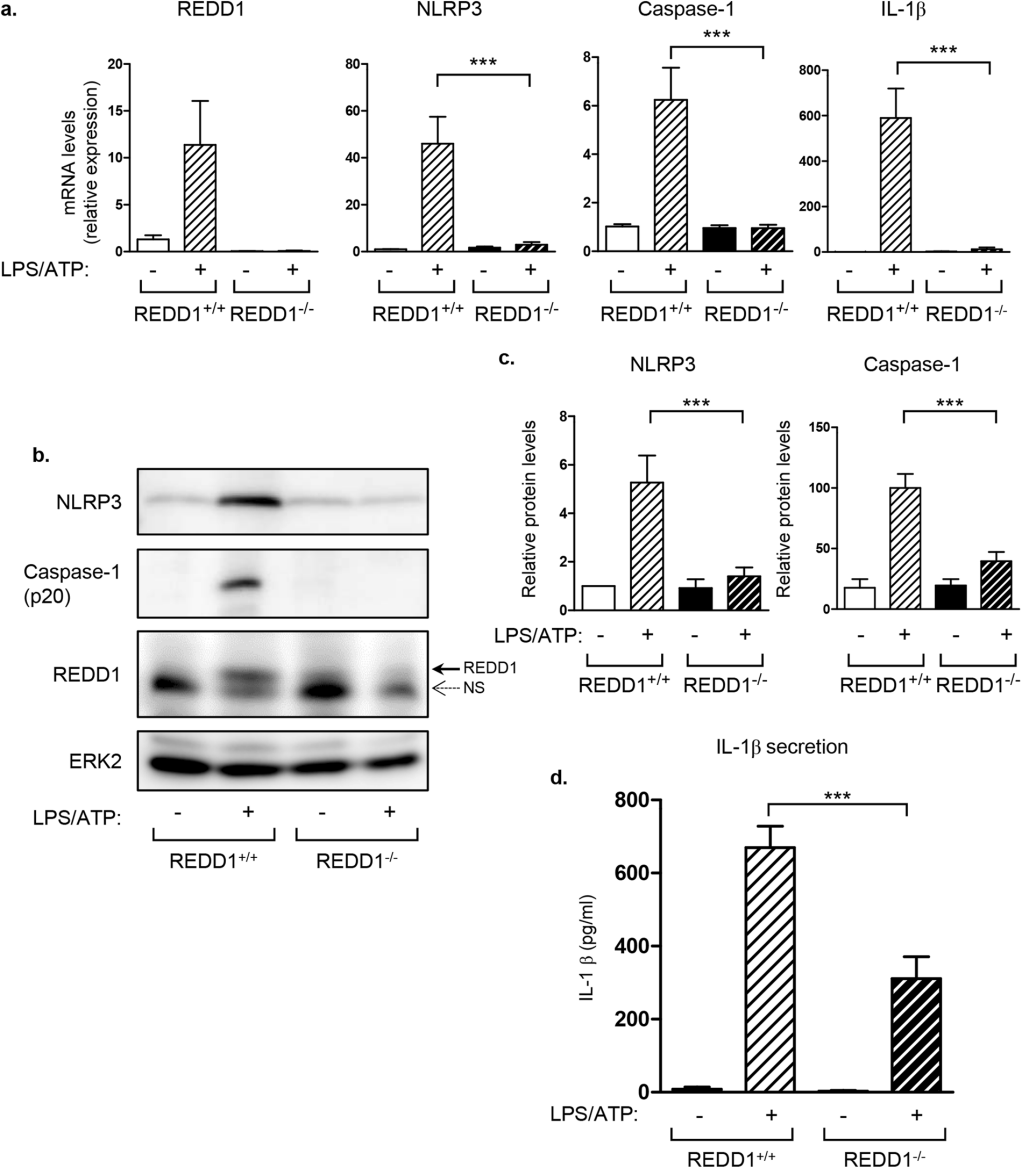
Absence of REDD1 inhibited the activation of NLRP3 inflammasome in bone marrow derived macrophages in response to LPS and ATP. Bone marrow derived macrophages (BMDM) obtained from REDD1^+/+^ and REDD1^−/−^ mice were stimulated for 5 hours with LPS (100 ng/ml) followed by a treatment with ATP (5 mM) for 45 minutes. (**a**) mRNA expression was determined by quantitative RT-PCR (n = 5 independent experiments in duplicate). (**b**) Cell lysates were analyzed by immunoblots with indicated antibodies. (**c**) Quantification of relative expression of NLRP3 (fold of expression) and Caspase-1 (p20) (normalized to tubulin) with the value of REDD1^+/+^ treated with LPS taken as 100 is shown (n = 6–7 independent experiments). (**d**) IL-1β concentration was determined by elisa test in the culture supernatant (n = 5 independent experiments in triplicate). ***p < 0.0001.

Increased expression of proinflammatory cytokines in visceral adipose tissue under acute systemic inflammation happens primarily in macrophages from the stromal vascular fractions rather than in adipocytes^4^. We deciphered the mechanisms implicated in REDD1-induced regulation of inflammasome activation using bone marrow derived macrophages (BMDM) prepared from REDD1^+/+^ and REDD1^−/−^ mice. REDD1^+/+^ and REDD1^−/−^ BMDM were stimulated with LPS and ATP. In Fig. 3a, LPS/ATP treatment stimulated the expression of REDD1 mRNA, as well as the expression of NLRP3, caspase-1 and IL-1β mRNA. This induction of expression was significantly decreased in REDD1^−/−^ macrophages treated by LPS/ATP (Fig. 3a).

Assembly of the NLRP3 inflammasome complex leads to the activation and cleavage of caspase-1 into its mature form p20. LPS/ATP treatment increased REDD1, NLRP3 and caspase-1 (p20) protein expression in wild-type BMDM (Fig. 3b and c). In REDD1^−/−^ macrophages, LPS/ATP treatment was no longer able to induce NLRP3 expression and cleavage of caspase-1 (Fig. 3b and c). In REDD1^+/+^ macrophages, LPS/ATP stimulated IL-1β secretion, which was significantly decreased in REDD1^−/−^ BMDM (Fig. 3d). A similar result was obtained in J774 macrophages in which downregulation of REDD1 by siRNA transfection decreased NLRP3 expression after LPS and ATP stimulation (Fig. S1).

### Activation of p38 MAPK, JNK and NFκB was impaired in REDD1^−/−^ cells

**Figure 4:**
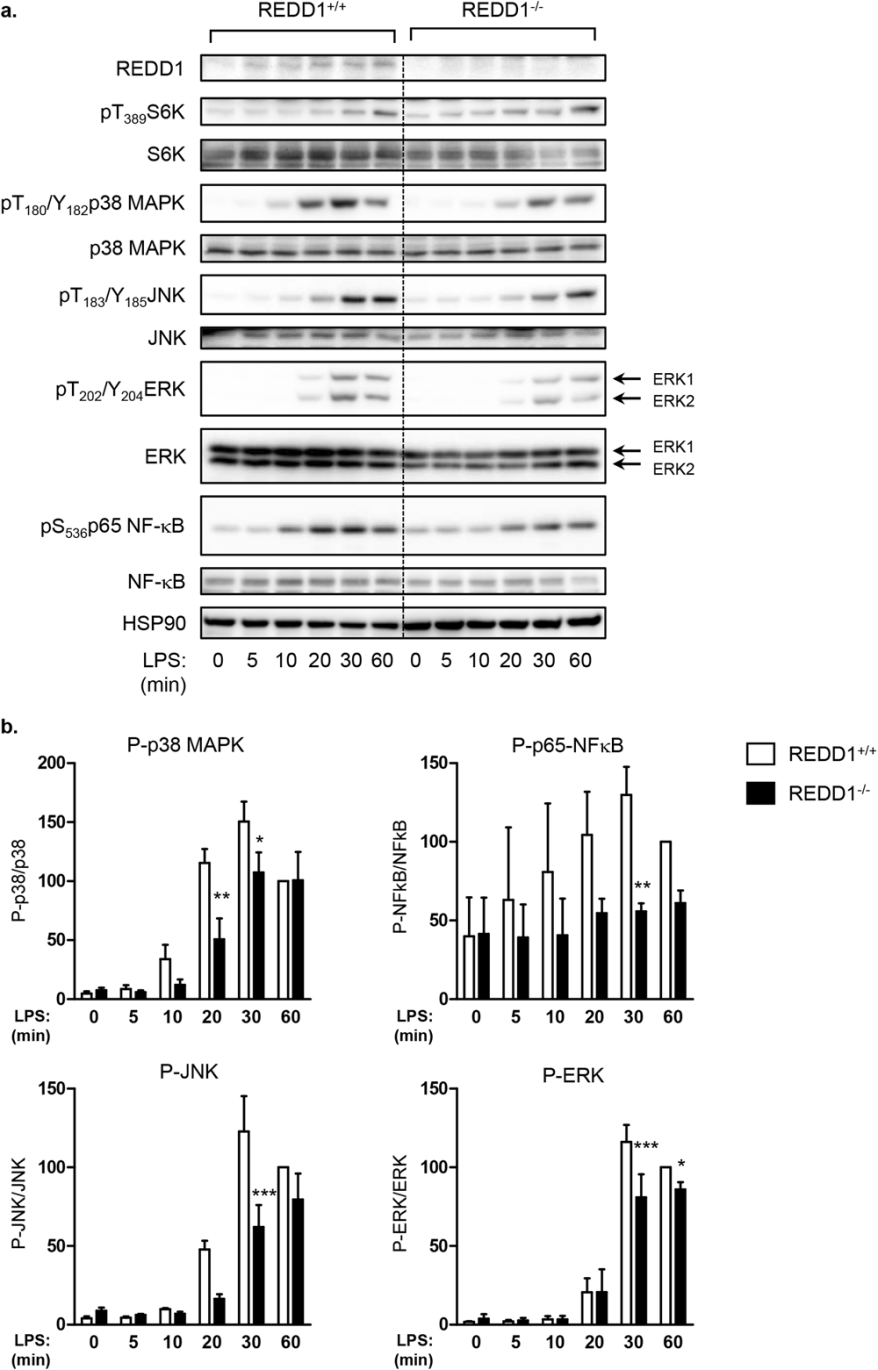
Activation of p38 MAPK, JNK and NF-κB was impaired in REDD1^−/−^ BMDM in response to LPS. REDD1^+/+^ and REDD1^−/−^ BMDM were stimulated with LPS (100 ng/ml) for indicated periods of time. Cell lysates were analyzed by immunoblots with indicated antibodies. (**a**,**b**) Quantification of phosphorylated proteins is shown with the value of REDD1^+/+^ treated with LPS for 60 min taken as 100 (n = 3 independent experiments, *p < 0.05; **p < 0.01, ***p < 0.0001).

The priming of NLRP3 inflammasome is regulated by signaling pathways such as NFκB which induces the expression of NLRP3 and pro-IL-1β. We investigated whether invalidation of REDD1 could impair the activation of upstream pathways such as MAPK and NFκB signaling pathways. REDD1^+/+^ and REDD1^−/−^ BMDM were stimulated for increased period of times with LPS (Fig. 4). LPS stimulated the expression of REDD1 as soon as few minutes of treatment. REDD1 has been described to act as an inhibitor of mTORC1. Indeed, in REDD1^−/−^ BMDM, phosphorylation of S6K, a substrate of mTORC1, was increased compared to wild-type macrophages (Fig. 4a). As expected, LPS stimulated the phosphorylation of p38MAPK, JNK, ERK1/2 and p65-NF-κB. In REDD1^−/−^ BMDM, the activation of p38 MAPK, JNK, ERK and NF-κB was significantly decreased after LPS treatment (Fig. 4a and b). The same pattern of activation was also observed in MEF (Fig. S2), since LPS and IL-1β were less potent to activate p38 MAPK, JNK and p65-NF-κB in MEF REDD1^−/−^ cells compared to wild-type MEF.

### Regulation of inflammatory pathways in REDD1^−/−^ cells was independent of mTORC1 activity

**Figure 5:**
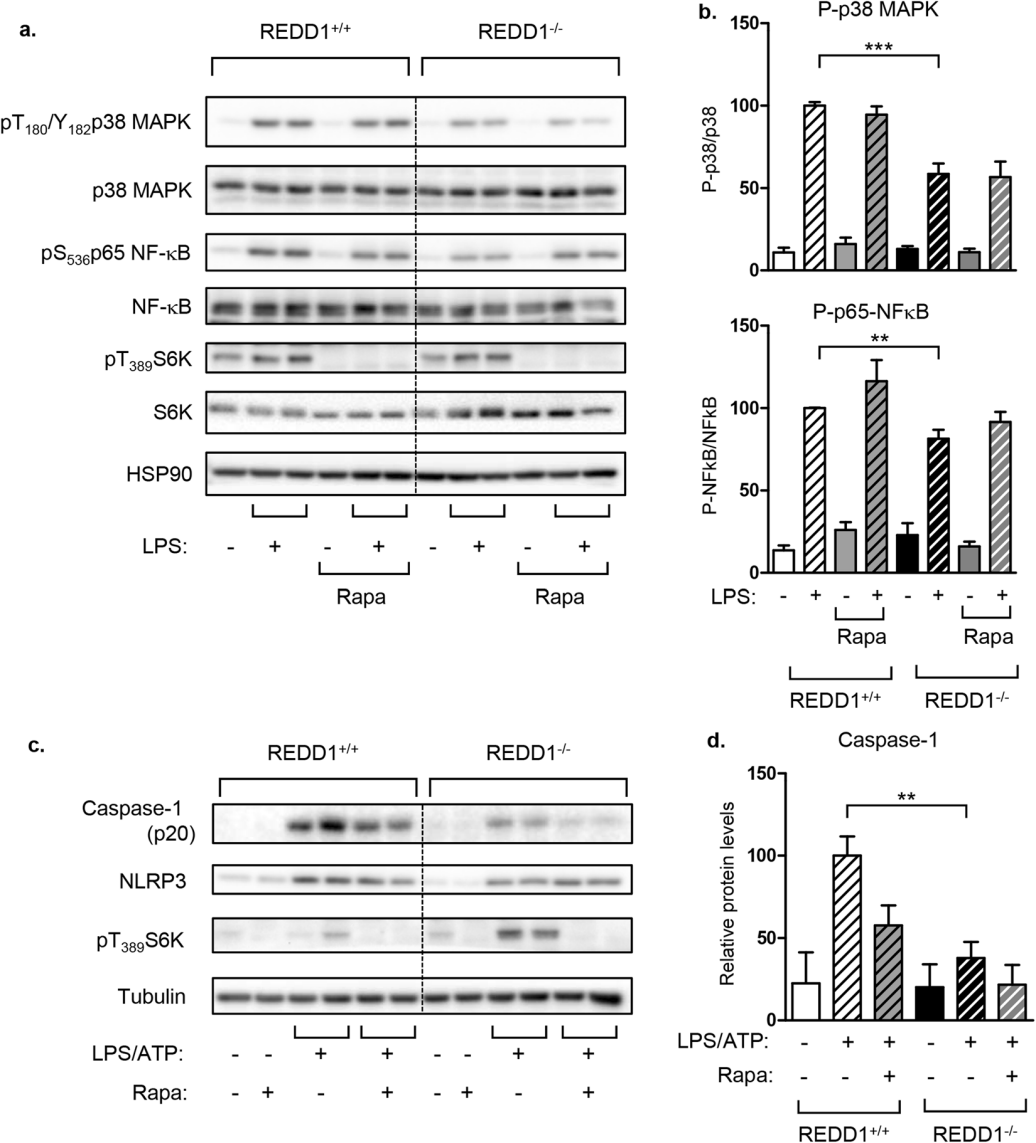
Inhibition of inflammation in REDD1^−/−^ BMDM was mTORC1 independent. (**a**) REDD1^+/+^ and REDD1^−/−^ BMDM were treated with rapamycin (Rapa) 40 nM for 45 minutes before being stimulated with LPS (100 ng/ml) for 20 minutes. Cell lysates were analyzed by immunoblots with indicated antibodies. (**b**) Quantification of phosphorylated proteins is shown (n = 3 independent experiments) with the value of REDD1^+/+^ treated with LPS taken as 100 (**c**) REDD1^+/+^ and REDD1^−/−^ BMDM were treated with rapamycin (Rapa) 40 nM for 45 minutes before being stimulated with LPS (100 ng/ml for 5 hours) and ATP (5 mM for 45 minutes). Cell lysates were analyzed by immunoblots with indicated antibodies. (**d**) Quantification of caspase-1 p20 normalized to tubulin is shown with the value of REDD1^+/+^ treated with LPS taken as 100 (n = 3 independent experiments).

Since REDD1 inhibits mTORC1, we determine whether the inhibition of signaling pathways detected in REDD1^−/−^ macrophages could be due to an increase of mTORC1 activity. To this end, we treated REDD1^+/+^ and REDD1^−/−^ macrophages with rapamycin, an inhibitor of mTORC1, prior to LPS stimulation. Phosphorylation of p38MAPK and NF-κB was significantly decreased in REDD1^−/−^ BMDM in response to LPS compared to REDD1^+/+^ BMDM (Fig. 5a and b). Inhibition of mTORC1, shown by the decrease of S6K phosphorylation, did not restore the phosphorylation status of p38MAPK and NF-κB (Fig. 5a and b). A similar observation is made in REDD1^+/+^ and REDD1^−/−^ MEF treated with IL-1β (Fig. S3). Then, we treated BMDM with rapamycin before stimulation with LPS and ATP and we evaluated the expression of mature form of caspase-1 (Fig. 5c). The expression of caspase-1 and NLRP3 was significantly decreased in REDD1^−/−^ BMDM compared to REDD1^+/+^ macrophages and was not reversed after mTORC1 inhibition (Fig. 5c and d). These results suggest that REDD1 silencing inhibited the activation of inflammatory pathways independently of mTORC1.

### Impaired inflammation activation in REDD1^−/−^ macrophages ameliorated insulin resistance

**Figure 6:**
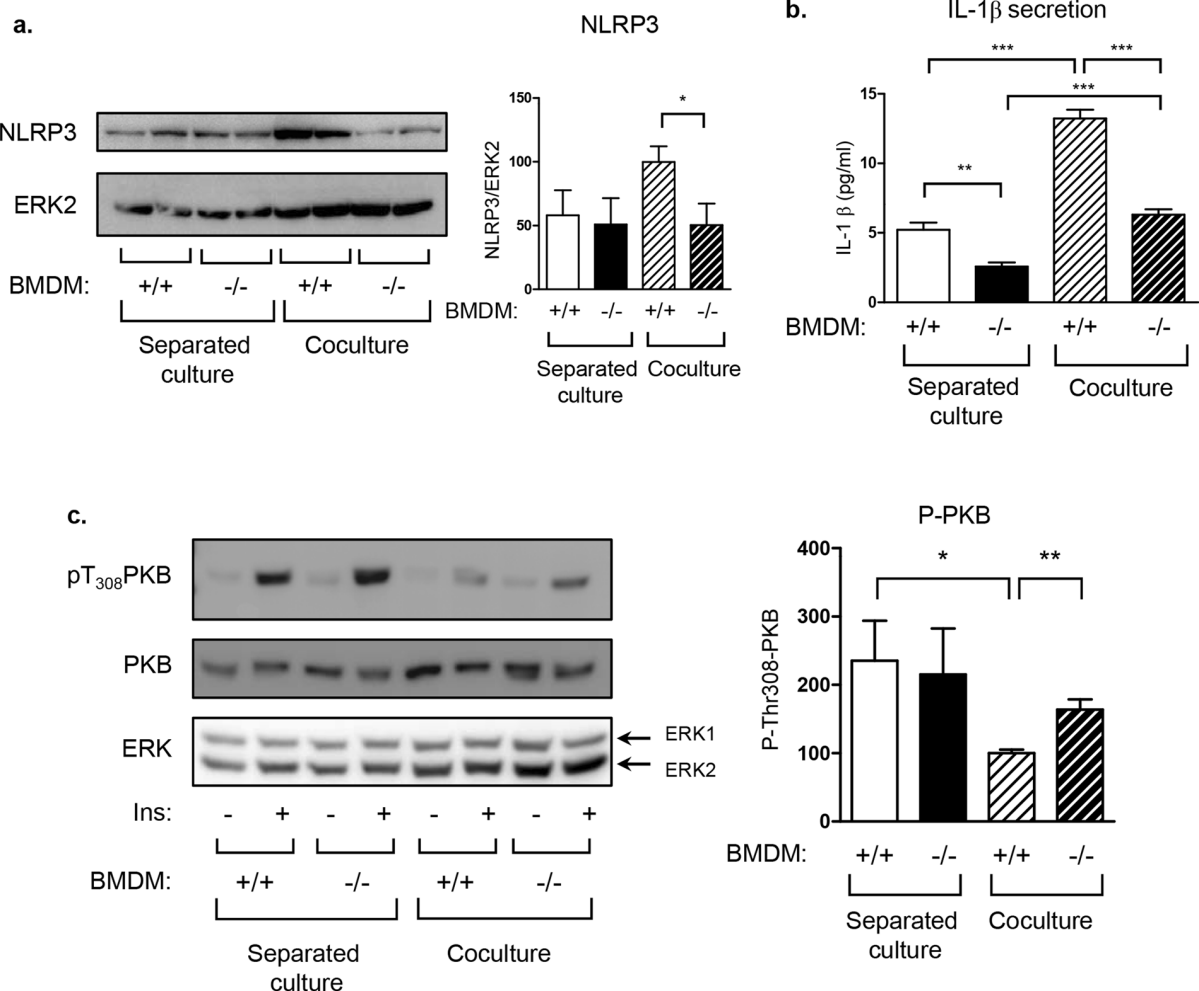
Invalidation of REDD1 in macrophages decreased IL-1β secretion in adipocyte-macrophage coculture. REDD1^+/+^ or REDD1^−/−^ BMDM were primed with LPS 100ng/ml for 3 hours before being cultured separetely or cocultured with 3T3-L1 adipocytes for 24 hours. (**a**) Cell lysates were analyzed by immunoblots with indicated antibodies. Quantification of NLRP3 (normalized to ERK2) is shown. (**b**) IL-1β concentration was determined by elisa test in the culture supernatant. (**c**) Cells were stimulated with insulin (1 nM) for 5 minutes and cell lysates were analyzed by immunoblots with indicated antibodies. Quantification of pT_308_ PKB with the value of coculture with BMDM REDD1^+/+^ treated with LPS taken as 100 (n = 4 independent experiments). *p < 0.05; **p < 0.01; ***p < 0.0001.

We studied the effect of absence of REDD1 expression in macrophages on the dialogue between adipocytes and macrophages. Interactions between macrophages and adipocytes can be recapitulated by coculture experiments. REDD1^+/+^ or REDD1^−/−^ BMDM were primed with LPS and seeded on 3T3-L1 adipocytes. As control, cells were cultured separately and cell lysates were mixed after harvest (Fig. 6). Coculture between 3T3-L1 adipocytes and REDD1^+/+^ BMDM induced an increase in NLRP3 expression, which was not detected in coculture between 3T3-L1 adipocytes and REDD1^−/−^ BMDM (Fig. 6a). Moreover, coculture between 3T3-L1 and wild-type BMDM stimulated IL-1β secretion compared to conditions in which adipocytes and BMDM were cultured separately (Fig. 6b). In coculture of 3T3-L1 adipocytes and REDD1^−/−^ BMDM, IL-1β secretion was significantly decreased, suggesting that REDD1 expression in macrophages regulated IL-1β expression in coculture (Fig. 6b).

In the adipose tissue, proinflammatory cytokines can participate to the establishment of an insulin resistance state by inhibiting insulin signaling pathway in adipocytes. REDD1^+/+^ or REDD1^−/−^ BMDM were primed with LPS and seeded on 3T3-L1 adipocytes. After 24 hours, coculture were stimulated with insulin. In separated cultures, adipocytes and BMDM were stimulated with insulin and cell lysates were mixed. Insulin signaling pathway was assessed by monitoring the phosphorylation of PKB (Fig. 6). In separated cultures, insulin stimulated PKB phosphorylation. When adipocytes were cocultured in presence of BMDM from wild-type mice, insulin-induced PKB phosphorylation was reduced by 57%. However, BMDM from REDD1^−/−^ animals were much less potent to induce such an insulin resistance state (Fig. 6c).

### REDD1 invalidation decreased oxidative stress

**Figure 7:**
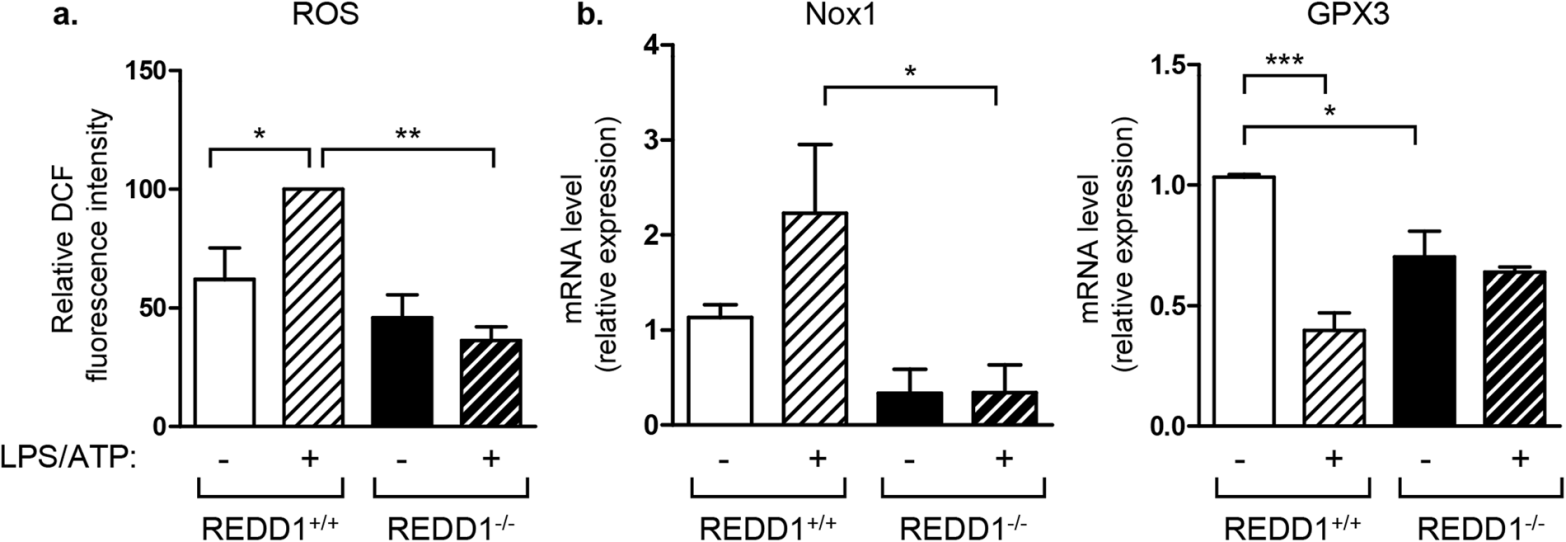
Oxidative stress was reduced in REDD1^−/−^ BMDM. REDD1^+/+^ and REDD1^−/−^ BMDM were stimulated for 5 hours with LPS (100 ng/ml) followed by a treatment with ATP (5 mM) for 45 minutes. (**a**) ROS production was measured by oxidation of DCFH-DA with the value of REDD1^+/+^ treated with LPS taken as 100. (**b**) mRNA expression was determined by quantitative RT-PCR (n = 3 independent experiments) * p < 0.05; **p < 0.01, ***p < 0.0001.

To understand molecular mechanisms involved in the regulation of inflammation by REDD1, we evaluated the level of oxidative stress, which is a potent activator of inflammation, in wild-type or REDD1^−/−^ macrophages. REDD1^+/+^ or REDD1^−/−^ BMDM were treated with LPS/ATP and reactive oxygen species (ROS) production was evaluated by measuring the intracellular H_2_O_2_. LPS/ATP stimulated the production of ROS in REDD1^+/+^ macrophages, whereas it failed to increase ROS production in REDD1^−/−^ BMDM (Fig. 7a). The production of ROS is modulated by opposing enzymes with oxidative (Nox-1, the NADPH oxidase) or antioxidative activities (glutathione peroxidase-3, GPx3). Nox-1 expression was increased in response to LPS treatment in wild-type BMDM, but not in REDD1^−/−^ cells. In wild-type BMDM, LPS significantly decreased GPx3 mRNA expression whereas GPx3 expression remained elevated in REDD1^−/−^ BMDM (Fig. 7b).

## Discussion

Inflammation is a protective response against harmful stimuli. It mobilizes the immune system and various other biological systems to eliminate the aggression and repair the tissue. In several pathological situations, inflammation is maintained and becomes damaging for the organism. Such chronic inflammation is found in several death-associated diseases. Various interconnecting signaling pathways are associated with the development of inflammation. Here, we provide evidence that REDD1, a stress-induced protein, is a key player in the control of inflammation.

We showed that REDD1 mRNA and protein expression were induced by LPS treatment in epidydimal adipose tissue, adipose tissue explants, macrophages and MEF. It is likely that this induction was mediated by p38 MAPK/MSK1 and COX-2/PGE2/PKA, two CREB-mediated mechanisms, as recently demonstrated in RAW264.7 and murine peritoneal macrophages^22^. However, we cannot ruled out that others transcription factors could be implicated in LPS-induced REDD1 expression, such as ATF4 or HIF-1 which are activated upon LPS treatment^23–25^. In addition, LPS or IL-1β rapidly increased REDD1 protein expression (Figs 4 and S2b), suggesting that REDD1 could be regulated at a post-translational level. Indeed, REDD1 can be regulated by proteasomal degradation after ubiquitination by E3 ubiquitin ligases including the CUL4A-DDB1-ROC1-β-TRCP E3 ubiquitin ligase complex, Parkin or HUWE1^26–28^.

Induction of REDD1 expression seems to play a crucial role in the activation of inflammation, since loss of REDD1 abrogates the expression of proinflammatory cytokines including IL-6, TNFα and IL-1β, and the activation of p38 MAPK, JNK and NF-κB by LPS. Moreover, NF-κB regulated-inflammatory genes such as NLRP3 and pro-IL-1β, as well as caspase-1 cleavage and IL-1β secretion are inhibited in REDD1^−/−^ tissues and cells. Thus, REDD1 would regulate the priming of NLRP3 inflammasome through NF-κB dependent pathway. Our results suggest that REDD1 would exert its action upstream NF-κB. Although REDD1 has been described as an inhibitor of mTORC1, our data do not illustrate a necessity for mTORC1 in the mechanisms by which REDD1 controls inflammation. This result was surprising since inhibition of mTORC1 reversed the anti-inflammatory phenotype in lung epithelial cells of REDD1^−/−^ mice exposed to cigarette smoke and LPS infusion^5, 21^. However, the description of a function of REDD1 independent of mTORC1 is not unprecedented. In particular, REDD1 has been shown to be involved in the regulation of autophagy independently of mTORC1^29^. Since oxidative stress is a well characterized modulator of inflammation, it is possible that the anti-inflammatory phenotype observed in REDD1^−/−^ macrophages would be due to a decreased amount of ROS. This reduction of ROS is associated with a decrease in the expression of the oxidative enzyme, Nox-1, and an increase in the expression of GPx3, a major scavenger of ROS. Nox-1 expression induced by LPS is regulated by a pathway involving IRAK-1/NF-κB cascade. The NF-κB inhibition in REDD1^−/−^ cells could explain the defect of Nox-1 expression^30^. On the other hand, LPS suppresses GPx3 expression by inhibiting the expression of nuclear receptors such as PPARα and PGC-1α^30^. Control of oxidative stress by REDD1 has already been reported in other models. Expression of heme oxygenase-1 (HO-1), a marker of oxidative stress, is decreased in lung of REDD1^−/−^ mice exposed to intratracheal LPS^5^. REDD1 has been localized, at least in part, in the mitochondria and shown to regulate ROS production^31^. REDD1 overexpression increases ROS production in fibroblasts invalidated for TP63, and absence of REDD1 induces mitochondrial dysfunction^7, 29^. Although it has been proposed that REDD1 could regulate ROS production through its association with the pro-oxidant protein TXNIP, we failed to detect any association between these proteins (data not shown). Other cellular mechanisms could also be involved since it has been recently shown that fatty acid metabolism regulates NLRP3 inflammasome activation through Nox4-dependent CPT1 activation^32^, and that REDD1 invalidation has been associated with a decrease in fatty acid oxidation in hypoxic tumor associated macrophages^33^.

The function of REDD1 in inflammation is supported by the observation that REDD1^−/−^ animals are protected from tobacco induced pulmonary injury^21^ or LPS infusion^5^. Here, we provide evidences that REDD1 could be involved in the control of insulin sensitivity *in vitro*. Obesity is characterized by chronic inflammatory state of the adipose tissue associated with an infiltration of the tissue by immune cells. In obese adipose tissue, macrophages secrete inflammatory molecules such as TNFα and IL-1β which are linked to the development of insulin resistance. Indeed, IL-1β induces insulin resistance by decreasing IRS-1 protein and inhibiting insulin-induced PKB phosphorylation^34^. The NLRP3 inflammasome activity promotes insulin resistance and genetic deficiency in NLRP3 inflammasome, ASC or caspase-1 ameliorates insulin signaling pathway in obese mice^35–37^. We show that absence of REDD1 in macrophages decreases the expression of NLRP3, secretion of IL-1β and development of insulin resistance in adipocyte-macrophage coculture. In conclusion, our study reveals the implication of REDD1 in the regulation of inflammation.

## Methods

### Materials

Insulin was obtained from Life Technologies (Saint Aubin, France), LPS ultrapure from InvivoGen (San Diego, USA) and IL-1β from PeproTech France (Neuilly sur seine, France). Antibodies were obtained from the following companies: REDD1 from Proteintech (Chicago, IL); pT_180_/Y_182_ p38 MAPK, p38 MAPK, JNK, pT_183_/Y_185_ JNK, pS_536_p65 NF-κB, IL1β, pT_308_PKB, PKB, ERK from Cell Signaling Technology (Beverly, MA); NFκB, HSP90 from Santa Cruz Biotechnology (Heidelberg, Germany); tubulin from Sigma-Aldrich; NLRP3, caspase-1 (p20) from Adipogen (San Diego, USA). The primer sets for real time PCR were purchased from Eurogentec (Seraing, Belgium) and Qiagen (Courtaboeuf, France). Culture media were obtained from Life Technologies (Saint Aubin, France). Rapamycin was obtained from Calbiochem (Nottingham, UK).

### Cell culture

REDD1^+/+^ and REDD1^−/−^ mouse embryonic fibroblasts (MEF) were obtained from L.W. Ellisen (Harvard Medical School, Boston, MA, USA) and were maintained in culture in DMEM containing 10% FCS (vol/vol). 3T3-L1 fibroblasts were obtained from ATCC (CL-173), and grown and induced to differentiate in adipocytes. 3 days after confluence, 3T3-L1 fibroblasts were treated for 2 days with DMEM–10% FCS (vol/vol) supplemented with isobutyl methylxanthine (250 nmol/l), dexamethasone (250 nmol/l), rosiglitazone (10 µmol/l) and insulin (800 nmol/l), and then for two additional days with DMEM–10% FCS containing 800 nmol/l insulin. Adipocytes were used between days 2 and 7 after the end of the differentiation protocol when the adipocyte phenotype appeared in more than 90% of the cells.

### Animals

Whole-body REDD1 null mice were generated by Lexicon Genetics Inc. (The Woodlands,TX) specifically for Quark Pharmaceuticals Inc. (Fremont, CA) and are the property of Quark Pharmaceuticals Inc. REDD1^+/+^ and REDD1^−/−^ mice were generated from C57BL/6 J heterozygous × heterozygous backcrosses (9 generations). Mice were housed in standard cages with free access to food and water under 12 h dark-light cycle. All animals were killed by cervical dislocation. The Principles of Laboratory Animal Care (NIH publication no. 85–23, revised 1985; http://grants1.nih.gov/grants/olaw/references/phspol.htm) were followed, as well the European Union guidelines on animal laboratory care (http://ec.europa.eu/ environment/chemicals/lab_animals/legislation_en.htm). All procedures were approved by the Animal Care Committee of the Faculty of Medicine of the Nice-Sophia Antipolis University, Nice, France and the French ministry of national education (#05116.02 and #201505&9143792_v2).

### Preparation of primary bone-marrow derived macrophages

Bone marrow cells were isolated from tibia and femur of 8–12 weeks old REDD1^+/+^ or REDD1^−/−^ mice. Mice were killed by cervical dislocation and the medullar cavity of bone was flushed with DMEM medium containing 30% of L-929 conditioned medium, 20% low endotoxin fetal bovine serum and streptomycin/penicillin/Fungizone (BMDM medium). Cells were seeded in 100 mm petri dishes and differentiated into bone marrow-derived macrophages in BMDM medium. The cells were ready to use around day 7 when the plates were semiconfluent.

### Coculture of 3T3-L1 adipocytes and macrophages

BMDM were primed for 3 hours with LPS (100 ng/ml) and seeded onto 6-well plates containing 3T3-L1 adipocytes. After 24 hours, coculture were stimulated with insulin (1 nM) for 5 minutes and cell lysate were prepared. As control, cells were cultured separately, treated exactly with the same condition and cell lysates were mixed after harvest.

### Injection of LPS

Male REDD1^+/+^ and REDD1^−/−^ littermates (12–14 weeks old) were injected with LPS (2 µg/g of body weight) dissolved in NaCl 0.9% or with NaCl 0.9% as control. After 5 hours, mice were sacrificed by cervical dislocation and epididymal adipose tissue was removed, frozen in liquid nitrogen and stored at −80 °C before mRNA and proteins preparation.

### Preparation of adipose tissue explants

Adipose tissue explants were prepared from epidydimal adipose tissue of REDD1^+/+^ and REDD1^−/−^ mice and incubated in DMEM containing 5% of heat inactivated SVF for 6 hours. Explants were treated with LPS (0.5 or 100 ng/ml) for 16 hours followed by a treatment with ATP (5 mM) for 45 minutes. After washes in PBS, adipose tissue explants were frozen in liquid nitrogen and stored at −80 °C before mRNA and proteins extractions.

### Western blot analysis

Serum-starved cells were treated with ligands, chilled to 4 °C, and washed with ice-cold phosphate-buffered saline (6 mmol/l Na_2_HPO_4_, 1 mmol/l KH_2_PO_4_, pH 7.4, 140 mmol/l NaCl, 3 mmol/l KCl) and solubilized with RIPA buffer (50 mmol/l Tris pH7.5, 150 mmol/l NaCl, 1% NP40, 0.1% SDS, 0.5% Na Deoxycholate, 1 mmol/l Orthovanadate, 5 mmol/l NaF, 2.5 mmol/l Na_4_P_2_O_7_ and Complete protease inhibitor cocktail (Roche Diagnostics, Meylan, France) for 30 min at 4 °C.

Epididymal fat pads were frozen in liquid nitrogen and stored at −80 °C until used. Tissues were solubilized using Precellys tissue homogenizer in ice-cold buffer containing 20 mmol/l Tris pH7.5, 150 mmol/l NaCl, 2 mmol/l Orthovanadate, 100 mmol/l NaF, 10 mmol/l Na_4_P_2_O_7_ and completed with 1% Triton X-100 and Complete protease inhibitor cocktail (Roche Diagnostics, Meylan, France).

Lysates were centrifuged (14,000 rpm) for 10 min at 4 °C, and the protein concentration was determined using BCA protein assay reagent (Thermo Fisher Scientific, Brebières, France). Cell lysates were analyzed by Western blot. Immunoblots were revealed using PXi4 GeneSys imaging system. Quantifications were realized using GeneTools or Fiji softwares^38^.

### Determination of IL-1β contents

IL-1β amount in cell culture media was determined by ELISA kit according manufacturer instructions (eBioscienceSAS, Mouse IL-1 beta Elisa Ready-SET-GO®).

### Measurement of Reactive Oxygen Species

Measurement was performed at 70–80% confluences using CM-H2DCFDA (5-(and 6) chloromethyl-2,7- dichlorodihydro-fluorescin diacetate, acetyl-ester; Invitrogen) in full medium at final concentrations of 10 µM for 45 minutes in the dark at 37 °C. After solubilization by sonification, ROS content was determined at excitation and emission wavelengths of 495 nm and 525 nm. ROS measurement was normalized with protein concentration determined using BCA protein assay reagent.

### Real-time quantitative PCR analysis

RNA was isolated from cells or epididymal adipose tissue (TRIZOL, Invitrogen), and cDNA was synthesised using Transcriptor first strand cDNA synthesis kit (Roche Diagnostics, Meylan, France). Real-time quantitative PCR was performed with sequence detection systems (StepOne, Applied Biosystems) and SYBR Green dye. Gene expression values were calculated based on the comparative cycle threshold Ct method (2^−ΔΔCt^). Levels of mRNA were normalized to the expression value of the housekeeping gene 36B4 and expressed relative to the mean of the group of controls. The primer sequence can be obtained upon request.

### Statistical analysis

Data are expressed as the mean +/− SEM. Statistical analysis was performed using GraphPad Prism. Differences among groups were compared using ANOVA with post-hoc analysis for multiple comparisons and Student’s *t* test when there were only two groups. *p* value < 0.05 is considered as significant.

## Electronic supplementary material

Supplementary Figures S1-S3
