# Supplementary material for: Implication of REDD1 in the activation of inflammatory pathways
Source: Sci Rep. 2017 Aug 1;7:7023. doi: 10.1038/s41598-017-07182-z (PMC5539207; doi:10.1038/s41598-017-07182-z)
Supplement: Supplementary file 1 — Supplementary Figures S1-S3 [file 41598_2017_7182_MOESM1_ESM.pdf]

Implication of REDD1 in the activation of inflammatory pathways

Faustine Pastor, Karine Dumas, Marie-Astrid Barthélémy, Claire Regazzetti, Noémie Druelle, Pascal Peraldi, Mireille Cormont, Jean-François Tanti, Sophie Giorgetti-Peraldi

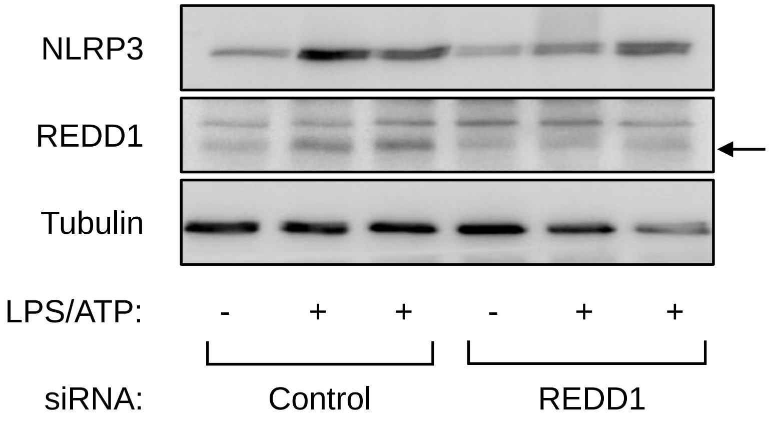

Supplementary Figure S1: J774 macrophages were transfected with siRNA against REDD1 and analyzed by immunoblots with indicated antibodies

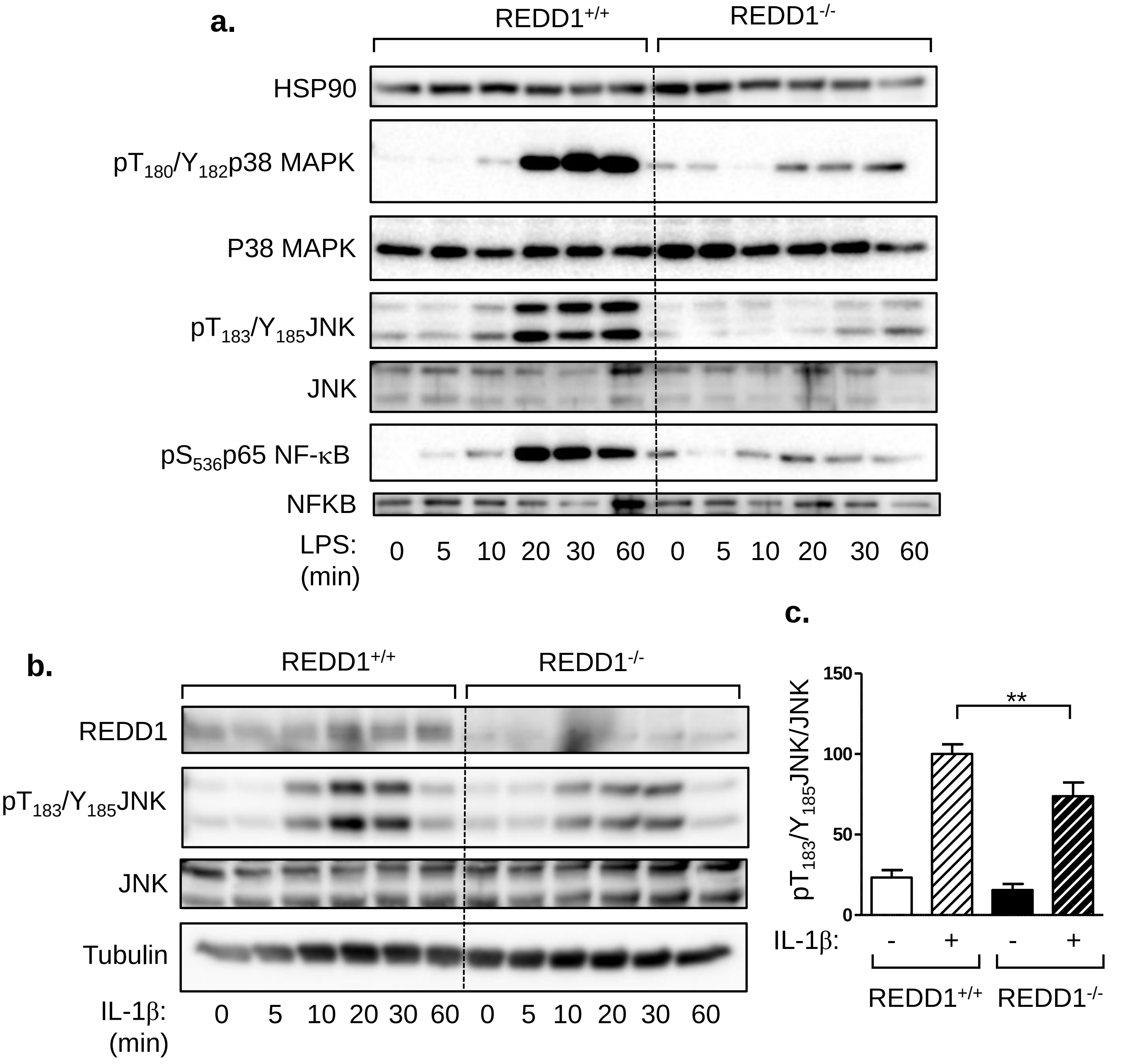

Supplementary Figure S2: Activation of p38 MAPK, JNK and NF-κB was impaired in MEF REDD1$^{-/-}$ in response to LPS and IL-1β

REDD1$^{+/+}$ and REDD1$^{-/-}$ MEF were stimulated with LPS (100 ng/ml) (a) or IL-1β (20 ng/ml) (b) for indicated periods of time. Cell lysates were analyzed by immunoblots with indicated antibodies. c: Quantification of pT$_{183}$/Y$_{185}$ JNK in response to IL-1β stimulation for 20 minutes (n=4 independent experiments in duplicate, ** $p<0.01$).

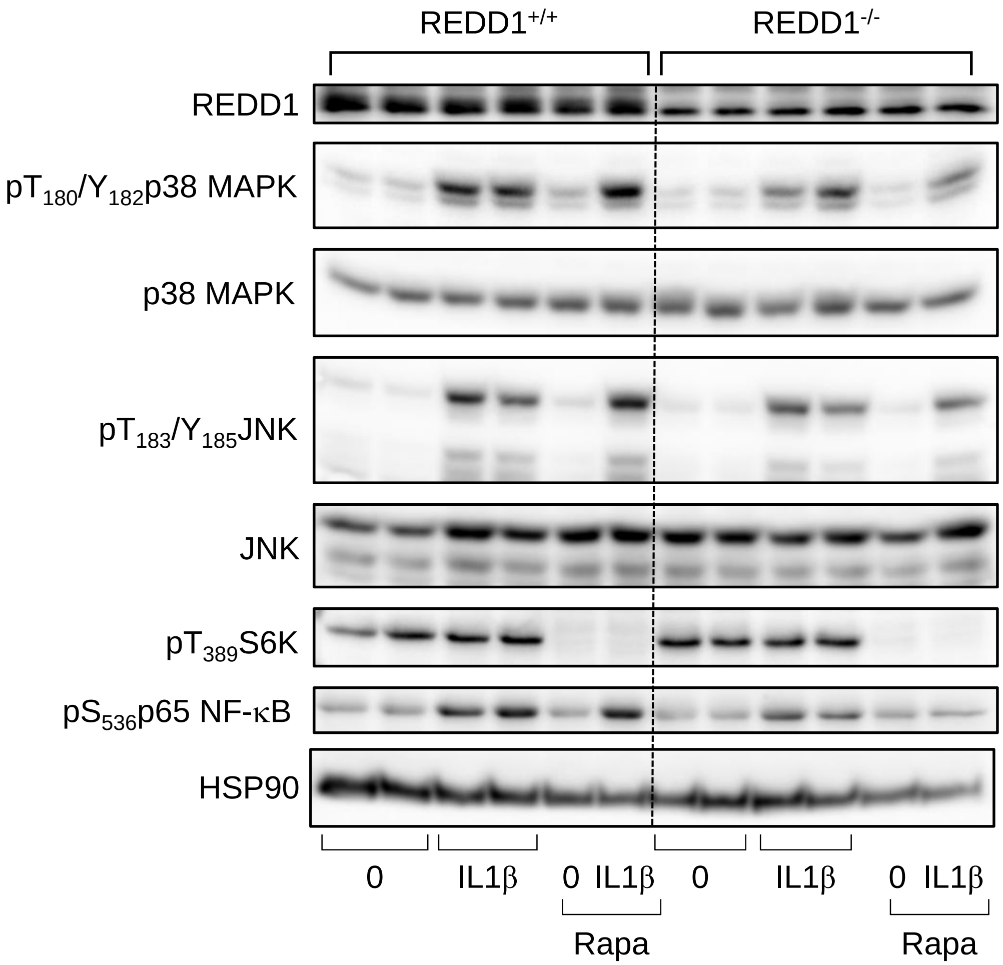

Supplementary Figure S3: Inhibition of inflammation in REDD1$^{-/-}$ cells is mTORC1 independent

REDD1$^{+/+}$ and REDD1$^{-/-}$ MEF were treated with rapamycin (rapa) 40 nM for 45 minutes before being stimulated with IL-1β for 20 minutes. Cell lysates were analyzed by immunoblots with indicated antibodies.
